# Use of Solubility Parameter to Design Dry Suspension of Cefaclor as a Dual Pack System

**DOI:** 10.4103/0250-474X.45399

**Published:** 2008

**Authors:** Kiran Kuksal, Kamla Pathak

**Affiliations:** Department of Pharmaceutics, Rajiv Academy for Pharmacy, NH No. 2, Mathura-281 001, India

**Keywords:** Solubility parameter, Cefaclor, Solubility, Solvent blends

## Abstract

One of the important methods to improve the solubility of a less water-soluble drug is by the use of co solvents. The solubility enhancement produced by two binary blends with a common co solvent (water-propylene glycol and propylene glycol-ethyl acetate) was studied against the solubility parameter of solvent blends (δ_1_) to evaluate the solubility parameter of drug (δ_2_). The binary blend water:propylene glycol (20:80) gave maximum solubility with an experimental δ_2_ value of 16.52 (Cal/cm^3^)^0.5^ that was comparable to the theoretical value of 16.52 (Cal/cm^3^)^0.5^ determined by molar volume method and 16.35 (Cal/cm^3^)^0.5^ when determined by method proposed by Lin and Nash. The solvent blend water:propylene glycol (20:80) in which the drug exhibited maximum solubility was used as the reconstituting medium for formulation of dry suspension of cefaclor. The percentage cumulative drug release of cefaclor from the formulation F7 was compared to the marketed formulation by calculating the *f*1 (dissimilarity factor) and *f*2 (similarity factor) factors. A higher *f*1 value and *f*2 value below 50 indicates difference between the two dissolution profiles.

Cefaclor, an orally active cephalosporin in clinical practice, belongs to the group of β-lactam antibiotics. It is a slightly water soluble drug and its antibacterial activity is dependent on the presence of β-lactam functionality that can be hydrolyzed under aqueous conditions^1^. Solubility and stability problems of cefaclor can be overcome by selection of a suitable co-solvent. One important concept of solubilization by co-solvents is the polarity scale, which includes surface tension, solubility parameter, dielectric constant and partition coefficient to express the polarity of the solvents^2^. The choice of an appropriate co-solvent is important to obtain maximum solubility of the drug and solubility parameter serves as a guide in the selection of appropriate co-solvent^3^,^4^.

The use of single mixture limits the polarity range while the binary mixture with a common co solvent allows to expand the polarity range and to test the influence of the co solvent on drug solubility^5^. Evaluation of solubility parameter in different solvent blends of various polarities provides an important insight into the solubility of drug. The aim of the present study is to determine the solubility parameter of cefaclor by evaluating the solubility of cefaclor in different blends of water:propylene glycol (PG) to overcome the problem of solubility and hydrolytic instability, and to design and formulate dry suspension for reconstitution of cefaclor as a dual pack system and compare with marketed formulation.

## MATERIALS AND METHODS

Cefaclor was obtained as gift sample from Siemens Laboratories, Gurgaon, India and Himedia dialysis membrane-50 was procured from Himedia Lab. Pvt. Ltd., Mumbai. Polyplasdone XL was a gift sample from ISP Technologies Inc., NJ. Methyl cellulose, microcrystalline cellulose, acacia gum and sodium citrate were obtained from Ranbaxy Fine Chemicals Ltd., New Delhi, and sodium benzoate, sodium starch glycolate, sucrose from Qualigens Fine Chemicals, Mumbai. The binary mixtures were prepared (by volume) with glycerin or propylene glycol (Ranbaxy Fine Chemicals Ltd., New Delhi) and all glass double distilled water.

### Solubility measurements:

Sealed flasks containing an excess of cefaclor in the pure solvents and solvent blends were shaken at 37±0.5° in a temperature controlled water bath (Hicon, India). When the saturation concentration was attained (after 72 h), the solid phase was removed by filtration through nylon filter disk (0.45 μ). The clear solutions were diluted with double distilled water and assayed in a double-beam spectrophotometer (Shimadzu Pharmaspec, UV-1700, Japan). The spectrophotometric measurements were performed at 264 nm. The densities of the solutions were determined at 37+0.5° in 10 ml pycnometer to convert molar solubility into mole fraction units^6^. All the experimental results are the average of at least three replicated experiments. The coefficient of variation (SD/mean×100) was within 2% among replicated samples for the solubility measurements.

### Solubility parameter determination:

Solubility parameter determination of cefaclor (δ_2_) was done by solubility measurement method (experimental method) and by theoretical methods namely molar volume method and by method proposed by Lin and Nash^6^. In solubility measurement method, the solubility parameter of cefaclor is assumed to be similar to that of the solubility parameter of the solvent (δ_1_) in which the drug exhibits maximum solubility^7^. Hence, the solubility data (Table 1) obtained by the method described in preceding section was used to determine δ_2_.

**TABLE 1:** SOLUBILITY OF THE BINARY MIXTURE BLENDS

Solvent blend Water:PG (%v/v)	δ_1_ (Cal/cm^3^)^0.5^	(δ_1_−δ_2_)	X_2_ (mg/ml)	Mole fraction solubility (X^i^_2_ × 10^−3^)
100:0	23.40	6.88	19.06	0.91
80:20	22.26	5.74	19.41	1.08
60:40	21.12	4.6	22.38	1.49
40:60	19.98	3.46	27.09	2.29
20:80	16.52	0.00	29.93	3.51
0:100	14.80	−1.72	14.08	2.68

The binary mixture blends, δ_1_ and δ_1_ − δ_2_ and the corresponding values of equilibrium experimental solubility and mole fraction solubility

The solubility parameter of cefaclor was determined by molar volume method by calculating the mole fraction solubility (X^i^_2_) of cefaclor in solvent blends containing water and propylene glycol in different ratios as shown in Table 1. The mole fraction solubility was calculated by using the following equation, ${{\text{X}}^{\text{i}}}_{2}={\text{n}}_{2}/{\text{n}}_{1}+{\text{n}}_{2}\left(1\right)$, where n_1_ = number of moles of solvent and n_2_ = number of moles of solute. A plot of mole fraction solubility of cefaclor in the various ratios of the binary mixtures was made against Δδ(δ_1_−δ_2_). The solubility parameter of the solvent blend (δ_1_) in which cefaclor showed peak mole fraction solubility represented the solubility parameter of cefaclor (δ_2_)^8^.

The method of Lin and Nash is based on the use of experimental mole fraction solubility of drug in given solvent blends. Thus δ_2_ can be determined by use of the following equation, ${\text{δ}}_{2}={\sum \text{X}}^{\text{i}}{\text{δ}}_{1}/{\sum {\text{X}}^{\text{i}}}_{2}\;\left(2\right)$, in which δ_2_ is the solubility parameter of cefaclor, X^i^_2_ is the mole fraction solubility of the solute in a given solvent and δ_1_ is the solubility parameter of the solvent^9^.

### Formulation of dry suspension of cefaclor for reconstitution:

Dry suspensions for reconstitution were prepared using the formulae shown in Table 2. All the ingredients were mixed in geometric proportion in a glass pestle-mortar and a sufficient volume of granulating agent (starch paste, 5% w/v for F1-F5 and alcohol 95% v/v for F6 and F7) was incorporated slowly. After enough cohesiveness was obtained, the mass was sieved though mesh #16. The granules were dried in oven (Jindal, Scientific Inst. Pvt. Ltd., India) at 60° for 30 min and the dried mass was passed through mesh #22. The dry products were reconstituted with reconstitution medium (water: propylene glycol, 20:80) for further evaluations.

**TABLE 2:** THE DIFFERENT FORMULATIONS OF CEFACLOR AND THEIR COMPOSITION

Ingredients (mg)	F1	F2	F3	F4	F5	F6	F7
Cefaclor	125	125	125	125	125	125	125
Sodium benzoate	15	15	15	15	15	15	15
Sucrose (g)	1.7	1.7	1.7	1.7	1.7	1.7	1.7
Colloidal silica	-	-	-	-	-	-	-
Acacia	250	250	250	250	250	250	250
Xanthan gum	-	-	-	-	-	-	-
Sodium citrate	75	75	75	75	75	75	75
Citric acid	50	50	50	50	50	50	50
Corn starch (5% aqueous paste)	q.s.	q.s.	q.s.	q.s.	q.s.	q.s.	q.s.
Methyl cellulose	125	250	500	-	-	-	-
Microcrystalline cellulose	-	-	-	250	500	-	-
Sodium starch glycolate	-	-	-	-	-	100	-
Polyplasdone XL	-	-	-	-	-	-	100
Color (orange)	q.s.	q.s.	q.s.	q.s.	q.s.	q.s.	q.s.
Flavor (orange)	q.s.	q.s.	q.s.	q.s.	q.s.	q.s.	q.s.

To be reconstituted to 5 ml with water: PG (20:80).

### Evaluation of reconstituted suspensions:

The pH of the reconstituted suspensions was measured using pH meter (DB-1011, HICON, India). The sedimentation volume (H_u_/H_o_) calculated against time was described in terms of the ratio of equilibrium settled height (H_U_) to original height (H_o_)^10^. The degree of flocculation was determined as the ratio of sedimentation volume of the flocculated suspension to the sedimentation volume of deflocculated suspension^11^. Ease of redispersion of suspensions was quantified by counting the number of strokes (given at an angle of 180°) required to redisperse the dispersed phase in 10 ml of the sample^12^. For single point viscosity determination of the reconstituted suspension, Brookfield viscometer (DV-II, Brookfield Eng. Lab. INC, USA), attached with spindle RV No. 4 was used. Suspension (75 ml) was taken in 100 ml beaker and the viscosity was measured at 100 rpm at room temperature. For drug content determination 1 ml of each suspension was dissolved in 100 ml of double distilled water, filtered, diluted as required and analyzed spectrophotometrically at 264 nm. Each value represents the mean content of three replicates^13^.

### *In vitro* drug release study:

Reconstituted suspension (5 ml) was taken in the donor compartment of lab fabricated glass diffusion cell (d= 2.4 cm) and 50 ml of double distilled water in the receptor compartment was stirred at 100 rpm and maintained at 37+0.5°. The HIMEDIA dialysis membrane-50 soaked in double distilled water for 24 h was used as a barrier membrane. Samples were withdrawn at different time intervals of 15, 30, 60, 90, 120, 150, 180, 240 and 360 min diluted with double distilled water and analyzed spectrophotometrically at 264 nm with reference to suitably constructed calibration curve. Release study was performed in triplicate. Marketed formulation (Keflor^®^, Ranbaxy, India) was also evaluated for *in vitro* drug release and compared with the best formulation.

## RESULTS AND DISCUSSION

Solubility of cefaclor was evaluated in solvent blends containing water:PG for the determination of δ_2_ as the varying blends of these provided a range of 14.80 - 23.40 (Cal/cm^3^)^0.5^ of δ_1_. The peak solubility (X_2_) of 29.93 mg/ml for cefaclor was observed in a solvent blend of water: PG (20:80) with δ_1_ of 16.52 (Cal/cm^3^)^0.5^. Thus the solubility parameter for cefaclor can be defined as 16.52 (Cal/cm^3^)^0.5^ as according to the solubility measurement method, δ_2_ is that value of δ_1_ at which the drug exhibits maximum solubility. Table 1 lists the solvent blends, the Hildebrand solubility parameter (δ_1_) of the solvent blends and the experimentally determined solubilities (mg/ml) of cefaclor.

**Fig. 1:**
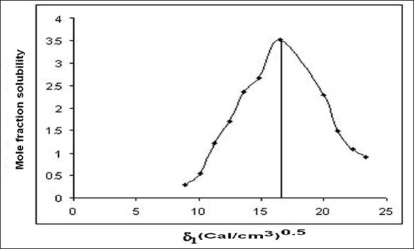
Solubility parameter versus mole fraction solubility profile of cefaclor by molar volume method

**Fig. 2:**
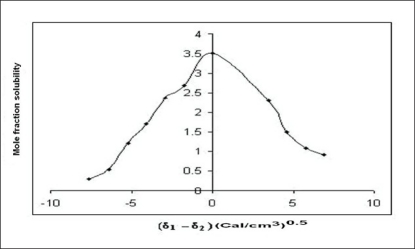
Mole fraction solubility versus (δ_1_-δ_2_) profile of cefaclor by molar volume method

The molar volume method was used to determine the peak mole fraction solubility of cefaclor in various solvent blends and the mole fraction solubilities X^i^_2_ of cefaclor and Δδ are tabulated in Table 1. Peak mole fraction solubility was determined to be 3.51×10^−3^ in solvent blend (water:PG, 20:80) with δ_1_ value 16.52 (Cal/cm^3^)^0.5^, which is in agreement with solubility measurement method. A plot of δ_1_ and X^i^_2_ (fig. 1) showed a bell shaped curve suggesting that both at lower and higher values δ_1_ = 16.52 (Cal/cm^3^)^0.5^ the solubility of cefaclor decreased. When Δδ was plotted against X^i^_2_ (fig. 2), the solubility parameter of cefaclor was confirmed at 16.52 (Cal/cm^3^)^0.5^ as it is that value of δ_1_ at which cefaclor exhibited peak mole fraction solubility and Δδ = 0. δ_2_ determined by the method of Lin and Nash were found to be 16.35 (cal/cm^3^)^0.5^, which is comparable to the value obtained by solubility measurement method and molar volume method.

Granular formulations of dry suspension for reconstitution were designed based on of two types of disintegrants, gel forming (methyl cellulose and sodium starch glycolate) and non-gel forming (microcrystalline cellulose and polypasdone XL) in order to assess their role on drug release (Table 2). Table 3 summarizes the physical and rheological characteristics of the reconstituted suspensions. The pH was found to be the range of 3.74−3.82, which is desirable for the stability of cefaclor^14^ and also accenuates palatability of the oral dosage form. The formulation F7, that showed maximum value (0.89 closest to 1 as compared to other formulations) of sedimentation volume and degree of flocculation is suggested to be homogenous in appearance and may not exhibit caking on long term storage. The viscosity of all the formulations ranged between 42–56 cps.

**TABLE 3:** COMPARATIVE PHYSICAL AND REHOLOGICAL PARAMETERS OF FORMULATIONS F1 - F7

Parameters		pH	Sedimentation volume	Degree of flocculation	Redispersibility (strokes)	Viscosity (cps)	Drug content (mg /ml)
F1	0 day	3.76	0.48	1.01	2	50	25.20
	At 7^th^ day	3.95	0.51	1.03	2	48	24.30
F2	0 day	3.77	0.60	1.08	2	54	25.17
	At 7^th^ day	3.90	0.62	1.10	2	50	24.69
F3	0 day	3.84	0.62	1.02	2	42	25.02
	At 7^th^ day	3.89	0.64	1.02	2	42	24.94
F4	0 day	3.81	0.66	1.05	2	48	24.06
	At 7^th^ day	3.92	0.67	1.07	2	45	24.69
F5	0 day	3.74	0.72	0.95	2	52	25.60
	At 7^th^ day	3.95	0.74	0.96	2	49	24.24
F6	0 day	3.78	0.77	1.07	2	58	25.09
	At 7^th^ day	3.87	0.79	1.03	2	55	23.02
F7	0 day	3.82	0.89	1.25	2	56	25.35
	At 7^th^ day	3.90	0.88	1.24	2	56	24.06

**Fig. 3:**
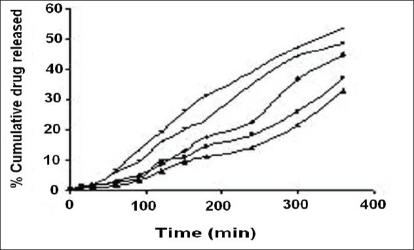
Release profiles of cefaclor from reconstituted suspension formulations containing methylcellulose F1 (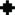), F2 (■), F3 (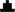) and microcrystalline cellulose F4 (+) and F5 (−)

**Fig. 4:**
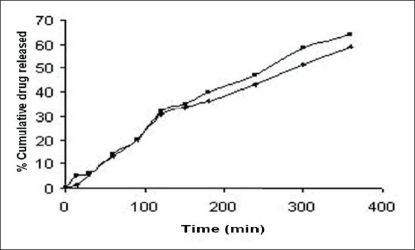
Release profiles of cefaclor from formulations containing sodium starch glycolate, F6 (+) and polyplasdone XL, F7 (■)

The release profiles of the freshly reconstituted suspensions are reported in figs. 3 and 4. The order of percentage cumulative drug release (%CDR) at 360 min is F7>F6>F5>F4>F1>F2>F3 with values of 64.14, 58.74, 53.48, 48.33, 44.50, 37.08, 32.51, respectively. In formulations F1, F2 and F3 a decrease in %CDR was observed as the concentration of gel forming disintegrants increased irrespective of the amount of the suspending agent used whereas in formulations F4 and F5, an increase in %CDR drug release was observed with increasing amounts of non-gel forming disintegrants. In an attempt to enhance the %CDR, granular formulations containing superdisintegrant were made by non-aqueous granulation. Formulation F6 containing sodium starch glycolate, the gel forming superdisintegrant showed lesser drug release when compared to F7 containing polyplasdone XL, both used in similar concentrations. The swelling of superdisintegrant by gel formation can delay dissolution, as the drug must diffuse through the gel layer before being released^15^. Highest drug release obtained with formulation F7 containing polyplasdone XL may be attributed to high cross-link density, swelling without gelling and thus not hindering the dissolution of drug.

Stability studies were accomplished at two levels, one for the reconstituted suspension for one week (Table 3) and other for the dried product and reconstituting medium for a period of three months (Table 4). No significant changes were observed after 3 months in physical and rheological characteristics of the suspension reconstituted with water: propylene glycol (20:80) as well as for the dried product including chemical stability (defined as maintenance of more than 80% of initial concentration) and drug release.

**TABLE 4:** STABILITY DATA OF DRY SUSPENSION AND RECONSTITUTING MEDIUM

Test	Parameters	Time (months)			
		
Sample	Evaluated	0	1	2	3
Dry	pH	3.85	3.79	3.77	3.77
Suspension	Drug content (mg/ml)	23.35	23.41	23.23	23.32
	% Cumulative drug release	64.14	64.55	63.40	63.78
Reconstituting Medium	Color change	-	-	-	-
	pH	4.95	4.90	4.93	4.98

**Fig 5:**
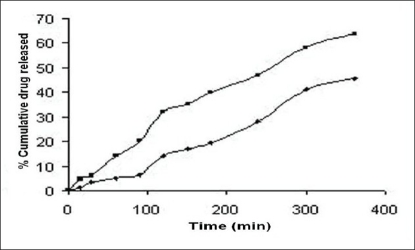
Release profiles of cefaclor from F7 (■) and marketed formulation, FM (+)

The %CDR of cefaclor from the selected reconstituted formulation F7 was compared with that of the marketed formulation FM and a higher release was obtained with F7 (64.14%) when compared to the marketed formulation FM (45.66%) (fig. 5). The *f*_1_ (dissimilarity factor) and *f*_2_ (similarity factor) factors were calculated to assess similarity of dissolution profiles of formulations. The *f*_1_ and *f*_2_ factors were equal to 43.62 and 17.65, respectively. A higher *f*_1_ and *f*_2_ value below 50 indicates difference between the two dissolution profiles^16^. Conclusively the solubility studies based on solubility parameter are useful indicator for selection of appropriate solvent blend for the formulation of a stable and efficacious liquid dosage form.
